# 

**Published:** 1900-04-14

**Authors:** 


### NOTICE TO CORRESPONDENTS.

All MS., Letters, Books for Review, and other matters intended for the Editor should be addressed The Editor, "The Hospital," The Hospital Building, 28 & 29, Southampton Street, Strand, W.C.

The Editor cannot undertake to return rejected MS., even when accompanied by stamped directed envelope.

### Notice to Advertisers and Subscribers.

All Advertisements, Orders for Copies of The Hospital, and Business Communications should be addressed to **THE MANAGER (Not to the Editor)**, The Hospital, The Hospital Building, 28 & 29, Southampton Street, Strand, London, W.C.

The Cost of Subscription, post free, is as follows:—Per week, 2½d.; per quarter, 2s. 8d.; per half-year, 5s. 3d.; per annum, 10s. 6d. Foreign:—Per annum, 15s. 2d., payable, in all cases, in advance.

**NOTICE.—Vol. XXVI. of "The Hospital" (April, 1899, to September, 1899), in handsome cloth binding, is now on sale at the Office of this Journal, price 7s. 6d. Cases for binding the Numbers contained in this Volume 1s. 6d. Index to Vol. XXVI., 2½d., post free.**

**The Monthly Part for April is now ready. Price 6d., by post 8d.**

### NOTES ON BOOKS.

The *City Press* C.I.V. War Souvenir is a publication of which three parts have now been issued, containing portraits of the members of the City Imperial Volunteers, together with illustrations of incidents in which they have been concerned. All who have friends in this force will refer to it with much interest.

Speaking of South African affairs, mention should be made of Who's Who at the War (Adam and Charles Black), which for the small sum of sixpence tells all about everyone out there.

Dr. Haywood, medical officer of health for Haydock, has issued in pamphlet form a reprint (from *Public Health*) of the paper he recently read before the Society of Medical Officers of Health, on the modification of the life-table for England and Wales, produced by excluding certain diseases or causes of mortality. Those interested in statistics will be glad to have this paper in its separate form.

Willing's Press Guide, 1900, is a book of constant utility to all who have to do with newspapers, containing as it does a complete alphabetical list of newspapers and periodicals, with their prices, days of publication, addresses, &c., and also lists classified according to the various special interests served and the towns in which they are published. A good deal of general information is also supplied.

### BOOKS RECEIVED.

John Wright and Co.

"The Medical Annual," 1900. Eighteenth year.

Skeffington and Son.

"Disenchantment of Nurse Dorothy; a Story of Hospital Life." By Florence Baxendale.

Bailliere, Tindall, and Cox.

"Mock Nurses of the Latest Fashion, A.D. 1900." By Frederick James Gant, F.R.C.S.

"Diphtheria: Being the Harben Lectures." By William R. Smith, M.D., D.Sc., F.R.S.Edin.

Adam and Charles Black.

"Plea for a Simpler Life," and "Fads of an Old Physician." By George S. Keith, M.D., LL.D., F.R.C.P.E.

S. W. Partridge and Co.

"Useful Work for Useful Hands." By J. T. Budd.

W. H. and L. Collingridge.

"City of London Directory for 1900."

Bailliere et Cie., Paris.

"Dictionnaire de Physiologie." Par Charles Richett.

The Medical Publishing Company.

"Heart Disease in Childhood and Youth." By Charles W. Chapman, M.D., M.R.C.P. With an introduction by Sir Samuel Wilks, Bart., M.D., F.R.S.

The University Press of Liverpool.

"Report of the Malaria Expedition of the Liverpool School of Tropical Medicine." By Donald Ross, D.P.H., M.R.C.S., H. E. Annett, M.D., D.P.H., and E. E. Austen.

"Instruction for the Prevention of Malarial Diseases."

**Periodicals and Pamphlets.**—Sunday at Home, Boy's Own Paper, Woman, Leisure Hour, Physician and Surgeon, Medical Record, Health, Medical Press.

Accouchement Requisites.

Ambulance Cases.

### NOW READY,

A REPRINT OF

### A NURSING HANDBOOK

TOGETHER WITH S. MAW, SON, AND THOMPSON'S

### COMPLETE CATALOGUE OF NURSING REQUISITES.

The above work is now republished at a cost of 1s. per copy. Messrs. S. M., S., and T. have been compelled to make this charge on account of the extreme popularity of the work and the great expense incurred in its production.

***Post Free on receipt of 1s.***

### S. MAW, SON, and THOMPSON,

7 to 12, ALDERSGATE STREET, LONDON, E.C.

### BOOKS ON NURSING.

**A COMPLETE SYSTEM OF NURSING.** Written by various contributors, consisting entirely of Medical Men and Nurses. Edited by HONNOR MORTEN, Author of "The Nurse's Dictionary," &c. 8vo., cl., 7s. 6d. *net.*

"We are bound to say that the greater part of this handy volume contains much that is useful and interesting. . . . Lastly, the list of recipes for invalid cookery is both original and serviceable."—*The Nurses' Journal.*

"As a book of reference, a nurse may find in its pages exactly the information she may want to resolve a doubt or difficulty."—*Glasgow Herald.*

"The chapter on 'The Nursing of Babies' is admirable, and should be read by all mothers as well as nurses."—*The Hospital.*

**WOUNDED IN WAR.** Surgeon's Handbook of Treatment. By F. ESMARCH, translated by H. H. CHILTON. With coloured plates and 536 engravings. 8vo., £1 8s.

**MANUAL OF FIRST AID.** Being a Text-book for Ambulance Classes and a Work of Reference for Domestic and General Use. By Dr. J. A. AUSTIN, Lecturer to St. John's Ambulance Association, and Author of "Ambulance Sermons." Illustrated with diagrams. Dedicated by permission to H.R.H. Princess Christian of Schleswig-Holstein. Crown 8vo., 3s. 6d. [*Ready.*

"Dr. Austin's little book is well and interestingly written. . . . The plan of the book follows the syllabus of the St. John's Ambulance Association, and is copiously illustrated with diagrammatic figures, which are plain and simple and accurate. Altogether the book is a good specimen of its class."—*Lancet.*

**HOW TO TREAT ACCIDENTS AND ILLNESSES.** A Handbook for the Home. By HONNOR MORTEN, Author of "Sketches of Hospital Life," &c. Second Edition. With illustrations. Post 8vo., boards, 2s.; cloth, 2s. 6d.

"A very sensible 'handbook for the home,' prepared by a practical person, presumably a lady with nursing experience. It tells what to do in case of common accidents, common emergencies, and the various common illnesses to which flesh is heir. The explanations are given in the simplest business-like language, and the incidental advice is full of common-sense, and tends to reassurance of the patient. To the systematic treatment of the various physical troubles liable to afflict a family are appended some useful general hints on nursing and on the preservation of health."—*Daily Chronicle.* "Just such a book as a nurse or a good housewife needs."—*Scotsman.*

**LYING-IN.** A Book for Every Mother. By MARIAN HUMFREY, of the British Lying-In Hospital, London, and Author of "A Manual of Obstetric Nursing." Small crown 8vo., paper covers, 1s.

DR. ALFRED HILL (Medical Officer of Health, Birmingham) says:—"I have gone through the book, and am convinced that if lying-in women will follow its directions, the discomforts, dangers, and diseases which too often attend the natural process will be either avoided or mitigated."

**1. A MANUAL OF OBSTETRIC NURSING.** By MARIAN HUMFREY. Part I.—MATERNAL. Part II.—INFANTILE. Crown 8vo., cloth, 3s. 6d. Dedicated by permission to H.R.H. the late Princess Mary of Cambridge, Duchess of Teck.

*The Hospital* says: "This volume is certainly a very valuable addition to the literature of obstetric nursing. Not only is every little detail mentioned, but its special excellence consists in the very lucid manner in which all is explained. . . . The arrangement of the chapters, and the advice given are beyond praise."

**2. A MANUAL OF OBSTETRIC NURSING.** By MARIAN HUMFREY. Vol. II. Part I.—MATERNAL. Part II.—INFANTILE. Crown 8vo., cloth, 3s. 6d. This volume treats of duties, special to the gravest emergencies, of Obstetric Nursing, and thus completes the curriculum of instruction.

LONDON: SAMPSON LOW, MARSTON & COMPANY, LIMITED., St. Dunstan's House, Fetter Lane, E.C.

